# An Intriguing Case of Rapidly Growing Vulval Fibroepithelial Polyp: Awareness of Histologic Mimickers is Crucial

**DOI:** 10.30699/ijp.2025.2044987.3372

**Published:** 2025-07-01

**Authors:** Aparna Jarathi, Seetu Palo, Poojitha Kanikaram

**Affiliations:** 1 *Department of Obstetrics and Gynaecology, All India Institute of Medical Sciences, Bibinagar, Hyderabad, India*; 2 *Department of Pathology and Laboratory Medicine, All India Institute of Medical Sciences, Bibinagar, Telangana, India*

**Dear Editor,**

Fibroepithelial polyps (FEPs), also known as fibroepithelial stromal polyps, are benign growths that arise from a combination of mesenchymal and ectodermal tissues. These lesions, while generally common—with an estimated prevalence of 46% in the general population—are rarely found on the vulva (1).

FEPs exhibit considerable variation in clinical presentation, ranging from small wart-like projections to large pedunculated tumors (2,3). They may appear as solitary or multiple lesions and can occur synchronously on both sides of the vulva. Although vulval FEPs are typically small (usually <5 mm), a few cases of unusually large lesions have been reported (1,2,4–6).

Herein, we report a case of a rapidly enlarging vulval FEP and discuss its clinicopathological mimickers that may complicate diagnosis. While the general presentation of vulval FEPs is well-documented, rapidly growing lesions of this nature are rare and underrepresented in the literature. Raising awareness about their atypical presentations and histologic mimics is essential for accurate diagnosis, timely intervention, avoidance of overtreatment, and improved patient outcomes.

A 37-year-old woman presented to the gynecology outpatient department with a rapidly enlarging swelling on the right side of the vulva, which had developed over ten days and was associated with mild to moderate discomfort. She reported no abnormal vaginal discharge, bleeding, or history suggestive of sexually transmitted infections or genital trauma. The patient was obese (BMI: 32.5 kg/m²), parous, and clinically stable with normal vital signs. General examination, including breast, thyroid, cardiovascular, respiratory, and abdominal systems, was unremarkable.

Local examination revealed a 5 × 4 cm pedunculated, firm, non-tender mass originating from the right labia majora. Pelvic examination findings were normal. Routine laboratory investigations were within normal limits. Pelvic ultrasonography showed no abnormalities except for a 3 × 3 cm intramural fundal fibroid. Elective surgical excision of the vulval mass was performed without complication. The mass was completely excised, and the patient’s postoperative recovery was uneventful.

Histopathological examination demonstrated a polypoidal lesion lined by partially ulcerated epidermis. The subepidermal region contained thin-walled, dilated blood vessels, scattered fibroblasts, and stromal cells embedded within fibrocollagenous stroma—findings consistent with a diagnosis of vulval fibroepithelial polyp. Mitotic figures were sparse and non-atypical (Fig. 1).

Similar to the case discussed, vulval fibroepithelial polyps (FEPs) are benign tumors commonly encountered in young to middle-aged women. They may also occur in premenopausal women undergoing hormone replacement therapy (5). Although the exact etiology remains unclear, hormonal stimulation and chronic inflammatory processes are considered plausible contributing factors (4). These lesions are thought to arise from specialized stromal cells within the female genital tract. Obesity and insulin resistance have also been proposed as potential stimuli for FEP growth (7). A few studies have reported an association between vulval FEPs and psoriasis (3); however, further investigation is needed to substantiate this link through more extensive clinical and pathological studies.

In the present case, an incidental uterine leiomyoma was detected via imaging. A similar coexistence of vulval FEP and uterine fibroid was reported by Kurniawati et al in a young woman presenting with primary infertility (1).

**Fig. 1:**
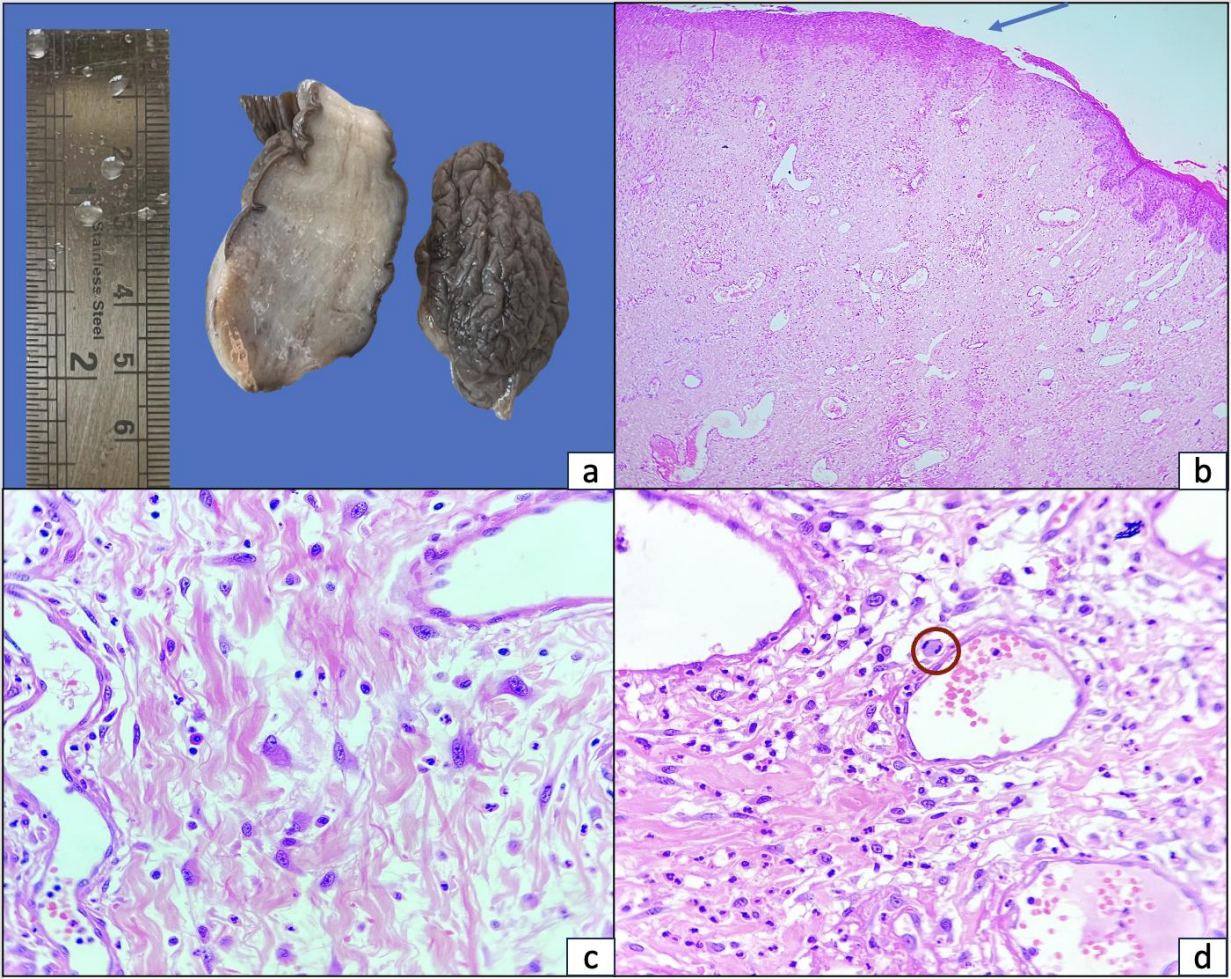
(a) Excised polypoidal tissue with gray-white, homogenously solid cut surface; (b) Microphotograph showing polypoidal tissue lined partly ulcerated epidermis (arrow) with subepithelium comprising of thin-walled, ectatic blood vessels along with scattered fibroblasts and stromal cells on a fibro-collagenous stroma [H&E stain, 40×]; (c,d) Higher magnification shows mildly edematous and paucicellular fibro-collagenous stroma with discrete plump stromal cells and occasional typical mitosis (circled in 'd') [H&E stain, 400×]

FEPs are typically small, asymptomatic, and slow-growing tumors by nature (6–8). In contrast, the patient in the current case presented with a lesion that grew rapidly—reaching 5 cm in just a few days—an uncommon occurrence that has been rarely documented. The patient’s obesity (BMI: 32.5 kg/m²) may have contributed to the lesion’s growth, as adiposity is known to induce hormonal imbalances and local irritation. Gupta et al reported a similar case in which a vulval FEP grew to 4 cm within 15 days (9). In such rapidly enlarging lesions, underlying malignancy must be considered.

The accelerated growth in this case posed a clinical challenge, raising concern for a possible neoplastic process. However, it is essential to recognize that rapid enlargement does not necessarily imply malignancy. Surgical excision followed by histopathological evaluation remains the gold standard for diagnosis and management. Imaging modalities such as computed tomography (CT) or magnetic resonance imaging (MRI) can be useful in preoperative assessment, particularly in determining the lesion’s origin, extent, vascularity, and internal composition (8,10). Typically, these masses appear hypointense on T1-weighted and hyperintense on T2-weighted MRI scans (6). According to Kato et al, characteristic features of FEP on MRI include hypointense zones surrounded by patchy hyperintense areas on T2-weighted images, and hyperintensity on T1-weighted sequences (11). As a more cost-effective option, ultrasonography can help define the lesion’s margins, depth, and predominant solid or cystic components (10). However, imaging findings may overlap with those of aggressive angiomyxoma, angiomyofibroblastoma, and cellular angiofibroma, limiting the specificity of radiologic diagnosis (11). Therefore, histopathological examination remains the definitive tool for diagnosis and differentiation from malignant mimickers.

The histologic features of FEPs are usually straightforward and seldom require ancillary studies such as immunohistochemistry. Microscopically, these polyps are hypocellular, composed of bland stellate stromal cells within a variably edematous fibromyxoid stroma. A mild inflammatory infiltrate may be present. The overlying epithelium may appear normal, atrophic, or focally ulcerated, as observed in the present case. Rarely, FEPs may exhibit stromal hypercellularity, cytologic atypia, or increased mitotic activity, which can lead to diagnostic confusion (12). Norris and Taylor, in their original description of vulvovaginal FEPs in 1966, highlighted the presence of atypical stromal cells and stressed the importance of avoiding misinterpretation of such features as malignancy (13).

**Table 1:** Histopathological features of site-specific vulval mesenchymal lesions

	Fibroepithelial Polyp	Vulval Fibroma	Cellular Angiofibroma	Superficial Angiomyxoma	Aggressive Angiomyxoma	Angiomyo-fibroblastoma
Location	Dermal based and/or subcutaneous, Usually polypoidal and exophytic	Dermal based and/or subcutaneous,	Subcutaneous	Dermal based and/or subcutaneous, may be polypoidal	Deep-seated	Subcutaneous
Margins	Usually merges with the normal surrounding tissue, no Grenz zone	Usually merges with the normal surrounding tissue	Circumscribed	Circumscribed	Infiltrative margins	Circumscribed
Cellularity	Variable, usually hypocellular	Pauci-cellular	Cellular	Pauci-cellular	Pauci-cellular	Alternating hypo- and hyper- cellular areas
Myxoid areas	Present	Rarely seen	Rarely seen	Present	Present	Present
Blood vessels	Thin-walled ectatic blood vessels	Thick-walled	Small to medium calibre, thick-walled, hyalinised	Thin-walled	Medium to large calibre, thick-walled	Numerous, thin-walled capillaries
Mitotic activity	Sparse	Sparse	Variable, may be brisk	Sparse	Sparse	Sparse
IHC	ER+, PR+, desmin+, SMA -/+	CD34+, ER – , PR – , desmin –	ER+, PR+,	ER – , PR –, CD34+ (weak)	ER+, PR+, desmin+,	ER+, PR+, desmin+,BCL2+, CD99+
Molecular characteristics	---	---	Loss of 13q14 (FOX1A1)	---	HMGA2 gene fusion	---

The site-specific histological differential diagnoses of vulval FEP include vulval fibroma, aggressive angiomyxoma, superficial angiomyxoma, angiomyo-fibroblastoma, and cellular angiofibroma (5,12,14,15), as summarized in Table 1. Recognizing these mimickers is essential, as some display aggressive behavior and require wider surgical excision. In this case, careful histopathological evaluation ruled out these differentials. For instance, aggressive angiomyxomas typically display infiltrative margins and higher cellularity, while angiomyofibroblastomas show alternating cellularity and perivascular cuffing—features absent in this lesion.

When necessary, additional histologic sections and immunohistochemical staining can resolve diagnostic uncertainty. Stromal cells in FEPs commonly express estrogen and progesterone receptors, desmin, and occasionally smooth muscle actin (4). In a recent study by Olson et al, desmin expression was observed in 23 of 25 cases (92%) of vaginal and vulval FEPs, with focal MyoD1 positivity in 40%, while all cases were negative for myogenin (12). Some FEPs—particularly those arising during pregnancy—may display atypical mesenchymal cells with mitotic activity, raising concerns for sarcoma botryoides, a variant of embryonal rhabdomyosarcoma, which typically shows diffuse myogenin positivity (15). In the current case, the diagnosis of FEP was supported by a dermal tumor epicenter, low cellularity, absence of cytologic atypia, and sparse mitotic activity.

Small FEPs can be treated with cryotherapy or electrocautery. However, larger or atypical lesions, such as in the present case, necessitate prompt and complete surgical excision. Recurrence may occur in cases of incomplete excision or in association with pregnancy or tamoxifen therapy (7). No recurrence was observed during the 1-year follow-up period in this patient.

In conclusion, this case contributes to the growing literature on vulval FEPs by emphasizing the need to consider them in the differential diagnosis of rapidly enlarging vulval masses. It also highlights the diagnostic challenges these lesions may pose and the importance of correlating clinical, radiologic, and histopathologic findings to guide optimal management.

## Prior presentation

An e-poster presentation titled “Fibroepithelial polyp from right Labia Majora: A Quirky Presentation” was presented at the 66th ALL INDIA CONGRESS OF OBSTETRICS AND GYNAECOLOGY (AICOG 24) in Hyderabad, Telangana, India.
